# Consistency of health-related quality of life among people living with HIV: Latent statetrait analysis

**DOI:** 10.1186/s12955-018-0929-4

**Published:** 2018-05-24

**Authors:** Marcin Rzeszutek, Ewa Gruszczyńska

**Affiliations:** 1 Faculty of Psychology, University of Warsaw, Stawki 5/7, 00-183 Warsaw, Poland; 2 Faculty of Psychology, University of Social Sciences and Humanities, Chodakowska 19/31, 03-815 Warsaw, Poland

**Keywords:** Health related quality of life, Personality, Latent state-trait analysis, HIV/AIDS

## Abstract

**Background:**

The aim of this longitudinal study was to examine the consistency of health-related quality of life (HRQoL) among people living with HIV (PLWH) by breaking down the variance of repeated HRQoL measures into trait, state, and method components and to test the stability of HRQoL over time. In addition, we wanted to examine whether HRQoL trait components are related to personality traits, while controlling for selected socio-medical variables.

**Methods:**

Three assessments were performed with a six-month lag on each assessment. Each participant filled out a World Health Organization (WHO) Quality of Life-BREF to assess HRQoL and a NEO-FFI to measure Big Five personality traits. Overall, 82 participants out of 141 (58.2% of the initial sample) participated in all the assessments.

**Results:**

The HRQoL among PLWH represented a stable trait to a somewhat greater extent than a situational variability, although the proportions were domain and time variant. More specifically, psychological domain appeared to be the most consistent, whereas social domain appeared to be the most prone to situational influences. The trait component of HRQoL was positively related to being in a relationship, being employed, and being extraverted, and negatively related to neuroticism, which altogether explained 26% of the trait variance.

**Conclusions:**

HRQoL among PLWH is rather distinct from personality and socio-medical data, which indicates its uniqueness in a clinical practise. Thus, there is a need for a more comprehensive assessment of HRQoL among this patient group to capture an additional source of variance in this important theoretical construct.

## Background

Although a massive body of literature exists on the concept of well-being, including psychological well-being (PWB) (e.g. [1–6]), many controversies still exist with respect to the definition, the dynamics, and the implications of PWB on various areas of individual and social functioning [7]. One of these unresolved research questions is whether PWB should be viewed as a relatively stable trait throughout a person’s life, or a trait subject to situational variability [8–12]. In other words, it is not clear how many variations in PWB are inherent in the person, and how many are tied to occasion-specific, external factors. The first conceptualization is in line with several “top-down” theories of well-being, indicating a specific, mainly hereditary “set-point” level of well-being characteristic of individuals [13–15]. Conversely, “bottom-up” theories of well-being contend that it changes over time and is tied to various life events (e.g. [16–18]). Resolving the aforementioned controversy is of great importance not only for the theory, but also for the practical application of research on PWB, specifically the implementation of interventions to enhance PWB [19]. Nevertheless, until now, studies in this area mainly have been focused on one aspect of PWB, namely satisfaction with life (SWL), providing a rather coherent picture of the relatively stable nature of this cognitive component of PWB over time [9, 11, 20, 21]. The temporal dynamics of other PWB components, including quality of life (QoL), are greatly underscored, referring especially to health-related quality of life (HRQoL) [22]. So far, previous research findings have been in favor of both a relatively consistent nature of HRQoL [23], as well as the notion that it may have some genetic basis [24] underlying its situational variability [25, 26]. According to Sprangers and Schwartz [22], these conflicting results may be attributed to the multidimensional character of HRQoL, which consists of both changeable (e.g., emotional functioning) and relatively stable (e.g., physical functioning) domains. Thus, investigating the proportion of state vs. trait variance in HRQoL may be crucial, not only from theoretical point of view, but also for implementing successful interventions tailored for a specific area of a patient’s functioning.

The aforementioned problem seems to be of special importance among people living with HIV (PLWH). On one hand, due to great advances in HIV treatment, HIV infection is now a chronic, manageable health problem [27, 28]. On the other hand, PLWH still struggle with intense HIV-related distress originating from a wide variety of psychosocial stressors [29–35]. In addition, PLWH are reporting significantly lower HRQoL, not only compared with the general population, but also in comparison with other chronic diseases [36]. The literature on HRQoL among PLWH is huge, but very inconclusive [36–39], indicating the varying impact that clinical, and psychosocial factors may have on HRQoL. Importantly, whereas previous studies have spotlighted the major role of clinical variables in HRQoL among PLWH (e.g. [40]), an increasing number of researchers recently have highlighted psychosocial factors that may even outweigh the significance of medical factors [37, 41, 42].

Numerous authors have shown that PWB is influenced greatly by personality traits [43–46]. More specifically, Steel et al. [47], in a meta-analytic review, found that the variance in PWB explained by personality traits may range from 39% to as much as 63%, which argument is used to support the hypothesis on the stability of PWB [48]. As far as PLWH are concerned, it was revealed that personality traits may be associated with some clinical variables, e.g., medication adherence (neuroticism negatively; [49]) and CD4 count (conscientiousness positively; [50]). However, the role of personality traits – e.g., neuroticism, extraversion, openness to experience, and conscientiousness - is especially profound for HRQoL in this patient group [49, 51–53]. Interestingly, Burgeous et al. [51] found that neuroticism had a strong impact on HRQoL that outweighed the role of health status, which is in line with a recent meta-analytic review conducted by Chan-Huang et al. [54], indicating that personality significantly affects HRQoL and that its effect is stronger than socio-demographic and medical variables.

## Current study

Since most studies on PWB, and HRQoL in particular, among PLWH were conducted using a cross-sectional design [38], little is known about individual differences in HRQoL dynamics in this patient group, particularly the proportion of state vs. trait variance in HRQoL. Therefore, we conducted a longitudinal study to decompose variance of the repeated HRQoL measure into trait, state, and method components, and to verify the stability of HRQoL over time among participants. In addition, we wanted to examine whether the HRQoL trait component is related to personality traits, while controlling for selected socio-medical factors. Since we did not have newly diagnosed patients, but instead had those who had been under treatment for some time (see Table 1), we expected that the proportion of trait variance in HRQoL would be higher compared with the proportion of state variance after separation from domain-specific method variance. Secondly, for the same reason, we expected that overall HRQoL among participants would be stable for 12 months. Finally, based on the top-down theory, we hypothesized that the HRQoL trait component would be more strongly related to personality traits (e.g., neuroticism, extraversion, openness to experience, and conscientiousness) than to socio-medical factors.

**Table 1:** Baseline Socio-Medical Characteristics of the Initial and Final Sample

Variable	Sample	
	Initial *N* = 141	Final *N* = 82
Gender		
Male	120 (85.1%)	70 (85.4%)
Female	21 (14.9%)	12 (14.6%)
Age in Years		
M ± SD (Range)	40.18 ± 10.24 (19–76)	40.50 ± 11.47 (21–76)
Stable relationship status		
Yes	84 (59.6%)	49 (59.8%)
No	57 (40.4%)	33 (40.2%)
Education		
Elementary/Secondary	61 (43.3%)	31 (37.7%)
University degree	80 (56.7%)	51 (62.3%)
Employment		
Full employment	99 (70.2%)	53 (64.6%)
Unemployment/Retirement	42 (29.2%)	29 (35.4%)
HIV/AIDS status		
HIV+ only	120 (85.1%)	48 (80.5%)
HIV/AIDS	21 (14.9%)	16 (19.5%)
HIV Infection Duration in Years		
M ± SD (Range)	7.34 ± 6.20 (1–30)	7.39 ± 5.72 (1–30)
Antiretroviral Treatment (ART) Duration in Years		
M ± SD (Range)	5.67 ± 5.10 (1–23)	5.76 ± 4.88 (1–21)
CD4 Count		
M ± SD (Range)	609.57 ± 240.90 (200–2000)	645.73 ± 256.23 (200–2000)

*M* Mean, *SD* Standard Deviation

## Method

### Procedure

Participants were recruited from patients at the outpatient clinic in the hospital of infectious diseases. After the informed consent was obtained, the participants completed a paper-and-pencil version of the inventories and participated in the study voluntary, as there was no remuneration for the participation. The study’s eligibility criteria were as follows: age 18 years or older, medically diagnosed as HIV-positive, and currently receiving medical care from the clinic where the study was performed. The exclusion criteria included having HIV-related cognitive disorders diagnosed by psychiatrists working at the hospital. This study was approved by the ethics committee of the Faculty of Psychology, University of Finance and Management in Warsaw.

### Measures

#### Health-related quality of life

Health-related quality of life (HRQoL) was assessed using the WHO Quality of Life-BREF (WHOQOL-BREF), developed under a WHO initiative to assess this construct cross-culturally [55]. WHOQOL-BREF consists of 26 items used to measure four domains: somatic health, psychological health, social relationships, and environment. Higher values indicate higher quality of life in each domain. Cronbach’s alpha coefficients for the current study ranged between .81 to .90 for somatic domains for T1, T2 and T3; ranged between .75 to .88 for psychological domain for T1, T2 and T3; ranged between .69 to 83 for social domain for T1, T2 and T3; ranged between .80 to .86 for environmental domain for T1, T2 and T3.

#### Personality dimensions

Personality traits were measured with the NEO-FFI questionnaire by Costa and McCrae [56]. NEO-FFI consists of 60 items (12 per trait), to which participants responded on a five-point scale, from *strongly disagree* to *strongly agree*. Five indices were obtained: neuroticism, extraversion, openness to experience, agreeableness, and conscientiousness. The higher scores of each indicate on higher level of each trait. The Cronbach’s alpha for the current study ranged for all traits from .76 to .82 at T1, .72 to .76 at T2 and .71 to .75 at T3.

### Data analysis

To verify the research hypotheses, a latent state-trait (LST) analysis was performed [57, 58]. The LST models have been used increasingly in analyses of longitudinal data to capture the within-time consistency vs. situation variability of individual differences of a particular variable over time. There are different versions of LTS models, but we applied the one described by Schermelleh-Engel et al. [59], in which a single trait is a second-order factor of state factors. Specifically, it was a single-construct model (HRQoL) with four indicators (HRQoL domains) measuring a latent trait repeatedly within six-month lags by three latent state variables. Also, as each domain was measured by a different part of the questionnaire, we added four method factors to capture a method-related variance. Thus, the LST model allowed for a breakdown of HRQoL variance into four parts: stable-trait variance, state-specific variance (expressed by latent state residuals), method variance, and error variance. It may help to specify how many variances of HRQoL among PLWH were explained by patient characteristics and how many by situation-specific fluctuations over time. The LST models differ from latent growth-curve (LGC) models, as these latter models may capture long-lasting and systematic changes within a particular variable over a long period of time. However, as our participants were not newly diagnosed patients, but had been infected and under treatment for some time already, we did not expect any significant systematic changes within HRQoL. Therefore, LST models were used instead of LGC models [58].

The IBM SPSS Statistics and AMOS, both version 24 [60], were used for data analysis, which consisted of three steps. The first step focused on testing measurement invariance [61] and calculating consistency, occasion specificity, and method specificity for each indicator [59], which provided information on indicator reliability. Next, latent mean changes were estimated to check whether HRQoL values changed over 12 months. Finally, individual values for trait factors were imputed and regressed on sociodemographic, clinical, and personality variables to answer the question of whether the HRQoL trait variable is related to other personal characteristics.

## Results

### Study sample

The first assessment was conducted during June and July 2016. A total of 141 patients agreed to take part in the study and provided their contact details (i.e., phone number and/or e-mail address). The second assessment was performed during January and February 2017. Out of 141 participants from the first assessment, 113 participated in the second assessment. The last assessment was performed during May and June 2017, with 82 participants remaining. Table 1 presents the socio-medical characteristics for both initial (*N* = 141) and final (*N* = 82) sample.

### Descriptive statistics and missing-data analysis

The studied variables are present in Table 2. Results within HRQoL domains are relatively stable within the time frame. All the variables have a univariate skewness and kurtosis below values described by West et al. [62] as potentially problematic for multivariate normal distribution required for the maximum likelihood (ML) estimation. The Little’s Missing Completely at Random (MCAR) Test (chi-square = 53.832, df = 50, *p* = .330) indicated that the missing data were missing completely at random (MCAR, [63]), including socio-medical characteristics. Thus, to avoid a reduction in the statistical power of the study, we used ML estimation, available in AMOS, to impute the missing data [64]. Next, further analyses were done for all the participants who took part in the study, i.e., *N* = 141.

**Table 2:** Descriptive Statistics of The Studied Variables

Variable	M	SD	Kurtosis	Skewness	Minimum	Maximum
WHO_Somatic T1	25.21	4.93	.80	−.68	7	34
WHO_Somatic T2	25.10	5.16	.35	−.60	10	34
WHO_Somatic T3	24.06	5.51	.43	−.59	7	35
WHO_Psychological T1	22.70	3.93	1.54	−.88	6	30
WHO_Psychological T2	22.51	3.94	1.13	−.79	7	30
WHO_Psychological T3	21.43	4.64	.15	−.69	7	29
WHO_Social T1	11.33	2.28	.13	−.47	4	15
WHO_Social T2	11.06	2.58	.69	−.79	3	15
WHO_Social T3	10.43	2.63	.49	−.68	3	15
WHO_Enviromental T1	30.46	5.30	2.82	−1.29	9	40
WHO_Enviromental T2	30.57	4.86	1.01	−.75	13	40
WHO_Enviromental T3	29.73	5.62	1.91	−.92	8	39
Neuroticims	25.77	7.09	−.24	.02	7	44
Extraversion	24.84	5.24	1.93	−.92	2	35
Openness to experience	23.94	5.90	.16	.06	6	37
Agreeableness	28.81	6.19	.54	−.18	7	45
Conscientiousness	27.43	5.13	1.09	−0,67	7	37

*T1* First Assessment (N = 141), *T2* Second Assessment (B = 113), *T3* Third Assessment (N = 82)

### Measurement invariance and variance decomposition

**Fig. 1:**
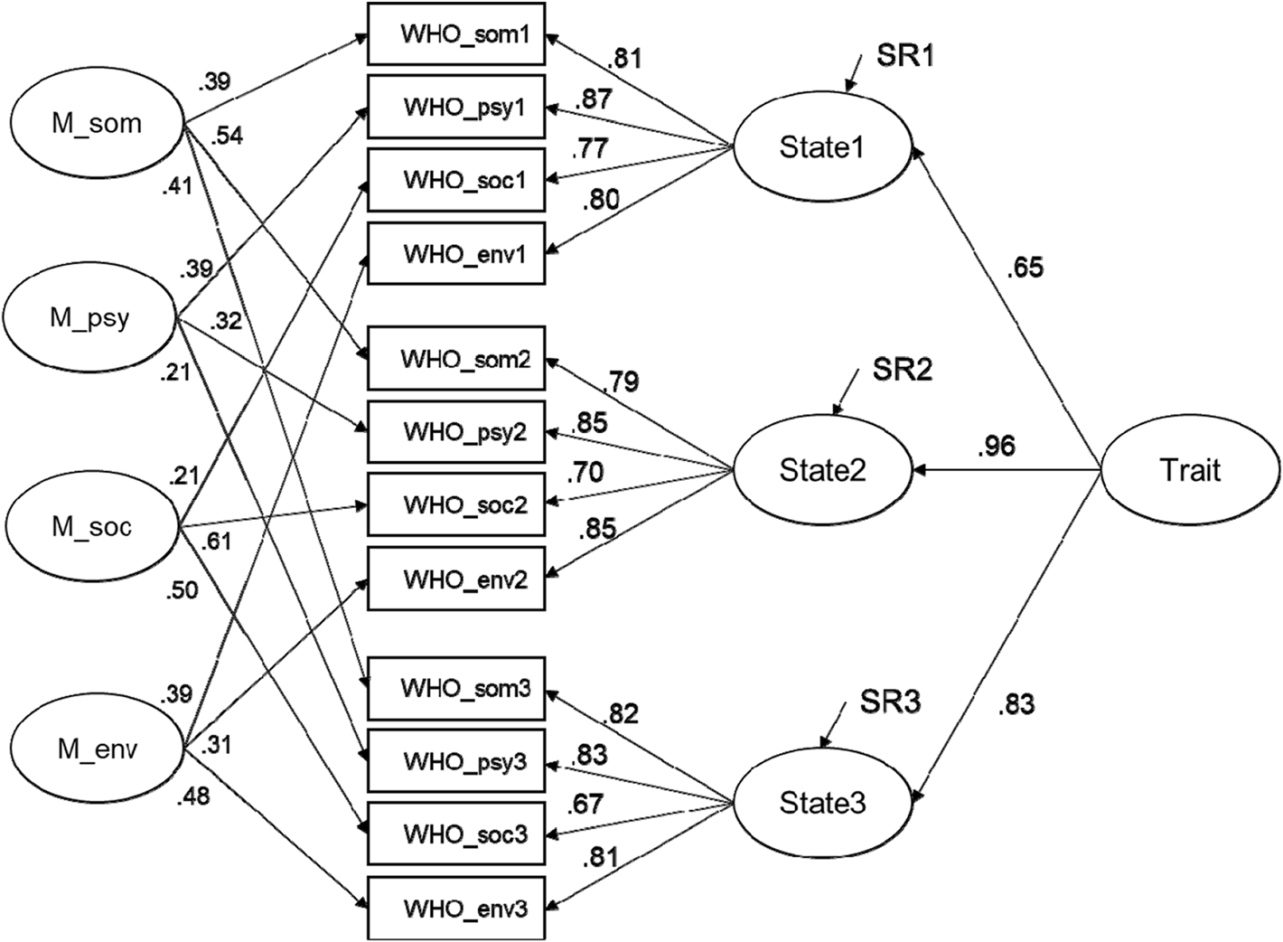
The final latent state-trait-method model for three measurement points. Reported are standardized loading parameter estimates. Error terms of domain indicators are removed from the figure for sake of clarity. SR- latent state residuals; M – method factors; som – somatic domain of WHOQOL-BREF; psy - psychological domain of WHOQOL-BREF; soc. - social domain of WHOQOL-BREF; env - environmental domain of WHOQOL-BREF. Numbers 1, 2, 3 depict consecutive measurement points

The goodness of fit of the model with configural invariance was satisfactory, χ^2^ (39) = 59.91, *p* = .02, χ^2^/df = 1.54, RMSEA = .06, 90% CI [.03, 0.9], CFI = .979, TLI = .957. Therefore, we checked whether the more constrained model with equal factor loadings of each domain variable on latent state variables fit significantly worse (weak factorial invariance). The comparison of models did not reject the assumption of weak factorial assumption (χ2 (6) = 8.58, *p* = .199). Thus, next in hierarchy model with strong factorial invariance was tested with the intercepts of each domain equal within time and it did not fit the data significantly worse (χ2 (8) = 9.33, *p* = .315; χ2 (53) = 77.81, *p* = .02, χ2/df = 1.47, RMSEA = .06, 90% CI [.03,0.8], CFI = .975, TLI = .962). It is presented in Fig. 1 as the final model, and variance components and reliability coefficients for its standardized solutions are provided in Table 3.

**Table 3:** Consistency, Occasion Specificity And Method Specificity For Each WHOQOL-BREF Domain

Indicator	Consistency	Occasion specificity	Method Specificity	Reliability	Error
Somatic Domain					
WHO_Somatic T1	.34	.47	.19	.82	.18
WHO_Somatic T2	.62	.06	.32	.92	.08
WHO_Somatic T3	.54	.25	.20	.84	.16
Psychological Domain					
WHO_Psychological T1	.35	.49	.16	.91	.09
WHO_Psychological T2	.80	.07	.12	.82	.18
WHO_Psychological T3	.64	.30	.06	.73	.27
Social Domain					
WHO_Social T1	.39	.54	.07	.64	.36
WHO_Social T2	.52	.05	.43	.86	.14
WHO_Social T3	.44	.20	.36	.69	.31
Enviromental Domain					
WHO_Enviromental T1	.34	.47	.19	.81	.19
WHO_Enviromental T2	.81	.08	.12	.81	.19
WHO_Enviromental T3	.50	.24	.26	.88	.12

*T1* First Assessment, *T2* Second Assessment, *T3* Third Assessment

In general, reliability is satisfactory, albeit excluding social domain. A precision of measurement within this domain should be regarded as doubtful due to the lowest values and high variability between measurement points. The average consistency is the highest for psychological domain (60%) and the lowest for social domain (45%). Thus, the domains seem to be differently prone to occasion-specific influences. Also, we observed that T1 and T3 values across domains are affected by situation or person and situation interactions (as they are indistinguishable in the LTS models [58]), whereas T2 scores are more strongly linked to stable disposition (52–81%), as well as method (12–43%). Taken together, this may suggest problematic homogeneity of domain indicators. The occasion-specific variability is the most pronounced for T1, as 47 to 54% of individual differences in domain scores is due to the context of measurement. It is also clearly visible at the state level: Stable personal disposition (“trait”) explains only 42% of inter-individual differences in the *overall* HRQoL at T1, as many as 91% at T2, and 68% at T3. Thus, we observed substantial variability in a variance structure decomposition between domains, as well as within time.

### Latent mean change

As the strong MI allows for mean comparison, for this purpose, the mean of the first latent state factor was fixed to be zero, whereas the means of the two remaining latent state factors were freed [58]. Consequently, the intercepts of those factors can be then interpreted as a difference relative to the first factor. For state at T2 (see Fig. 1), it was equal, − 0.131, and insignificant (*p* = .722), whereas for state at T3, it was equal − 0.993, and significant (*p* < .05). This indicates that although there was no change between T1 and T2, there was significant decrease in HRQoL level between T1 and T3.

### Time-invariant correlates of HRQoL trait component

A three-step hierarchical regression with stepwise method of variables entry (probability of F; criteria: entry = .5 and removal = .10) was used to establish HRQoL trait (HRQoL-T) correlates. In the first step, sociodemographic variables were introduced (gender, age, education, employment, and relationship status). In the second step, variables related to HIV infection (CD4 count, time since HIV diagnosis, duration of ARV treatment, and HIV/AIDS status) were entered. Finally, Big Five personality dimensions were included in the model. All the explanatory variables were measured at T1, and categorical variables were dummy-coded. Due to a number of potentially inter-related variables, collinearity was checked; VIF was below 1.2. The results are presented in Table 4. Among sociodemographic variables, only being in a stable relationship and being employed were significant, but weak-positive correlates of HRQoL-T. None of the clinical variables was significantly related to HRQoL-T. After controlling for sociodemographic and clinical variables, neuroticism and extraversion significantly added to the model in the third step of the regression analysis. Their coefficients have the opposite sign, but have very similar strengths. Altogether, the correlates explain 26% of HRQoL-T variance.

**Table 4:** Results Of Three-Step Hierarchical Regression Analysis With Stepwise Method of Variables Entry and WHOQOL-BREF Trait Component as Explained Variable

Model	F	df	ΔF	R^2^	Adjusted R^2^	Beta
Full Employment	13.36***	1; 139	–	.29	.08	.29***
Full Employment	11.13***	1; 138	8.20***	.37	.13	.24***
+ Stable Relationship						.23***
Full Employment	1.56***	1; 137	.8.24***	.43	.18	.20**
Stable Relationship						.21***
+ Extraversion						.23***
Full Employment	13.22***	1; 136	17.40***	.53	.26	.19**
Stable Relationship						.21**
Extraversion						.34***
+ Neuroticism						−.32***

*** *p* < .001; ** *p* < .01

## Discussion

The results of the study were partly in line with the first research hypothesis, i.e., we showed that HRQoL among PLWH represents a stable trait to a somewhat greater extent than a situational variability, although the proportions were domain and time variant. More specifically, we noticed differences between particular HRQoL domains, i.e., psychological domain appeared to be the most consistent, whereas social domain appeared to be the most prone to situational influences. Although several longitudinal studies have recently been conducted on HRQoL among PLWH (e.g. [37, 39]), none of them, to our best knowledge, has used the LST model. Thus, we do not have a direct benchmark to compare our results within this specific study design. However, our findings may have important theoretical and practical implications, as HRQoL is becoming a widely accepted patient-reported outcome in HIV research and counselling, as it may provide information that’s often difficult to obtain in clinical analysis [42]. Therefore, it is vital for HIV/AIDS health services to know which areas of HRQoL among PLWH should be addressed upfront in psychological interventions [65].

On one hand, consistency of psychological domain and occasion specificity of social aspects of HRQoL may suggest that the former is more person-rooted and the latter is a more situation-derived aspect of HRQoL. As such, they should be addressed differently in psychological interventions, first by person-oriented interventions (e.g., cognitive, behavioral interventions; [66]), and second by more interpersonal-focused techniques [67]. But other explanations also must be considered in the case of social domain of HRQoL. Namely, it was measured by the shortest three-item-only scale, which turned out to be unreliable enough among PLWH. Specifically, it includes a potentially sensitive item (i.e., *How satisfied are you with your sex life?*), which, in such a group, may measure different things during different stages in HIV patients’ lives, so the scores may be blurred by time-variant heterogeneity of the item interpretation, not by a situation itself. Thus, WHOQQL-BREF reliability of measurement calls for improvement in social domain, at least as far as PLWH are considered.

We also observed that HRQoL was relatively stable over time among participants, which was in line with our second hypothesis. Specifically, the aforementioned results also should be seen within the context of our sample, i.e., highly functional PLWH who have been undergoing antiretroviral therapy (ART) for some time already, and due to it have a mean CD4 count similar to that of the healthy population [68]. It also may shed some light on why clinical variables were unrelated to HRQoL among our participants, which has been noted in recent studies [41]. On a general level, this finding may be interpreted as a great advance in HIV/AIDS knowledge and treatment, as new advances have reduced HIV infections from a terminal and fatal disease to a chronic and manageable health condition [27]. It seems that nowadays, HIV infection does not entail serious psychological disturbances, as great progress in ART has provided opportunities for PLWH to live a longer life and has enabled successful adaptation to this disease [28].

Finally, personality traits, e.g., neuroticism and extraversion, appeared to be more strongly associated with trait components of HRQoL when compared with socio-medical data, which corresponded with our third hypothesis. Importantly, out of all the socio-medical data in our study, only being employed and being in a stable relationship were significantly related to the consistency of HRQoL, a finding that has been noted by other authors [69, 70]. In discussing this finding in the context of the trait-state conceptualization of well-being, it should be mentioned that these two personality traits predicted the highest proportion of stability of well-being among different study samples, even over a long period of time [14, 21]. Additionally, this finding is in line with the recent meta-analysis conducted by Chan-Huang et al. [54], who observed that personality outweighs the significance of socio-medical data in predicting HRQoL. Aforementioned results, therefore, are in line with HIV/AIDS literature on the role of personality traits and HRQoL. Neuroticism, in particular, predicted poorer HRQoL, mainly in psychological domains and independent of health status [51, 71]. Extraversion was positively related to HRQoL, especially within domains describing overall happiness and satisfaction from life, HIV mastery, and sexual functioning [49]. Nevertheless, existing studies are scarce and cross-sectional, so our research added to the literature by examining personality as a correlate of HRQoL consistency among PLWH in a longitudinal study design. Although these relationships are weak, which clearly implies a uniqueness of HRQoL measurement over personality assessment, clinicians perhaps should consider the role of personality in implementing successful psychosocial interventions to enhance HRQoL among PLWH. For instance, it was demonstrated that personality traits may impact HRQoL among different patient groups [54]. Specifically, personality indirectly changes HRQoL by influencing the process of coping with illness [72] and illness appraisal [73]. Several studies conducted among PLWH showed that coping and appraisal are crucial to the success of psychosocial interventions [65, 74, 75].

### Strengths and limitations

This study has a few strengths, namely longitudinal and theory-driven study design, as well as examination of consistency of HRQoL among a high-risk sample, i.e., PLWH. However, a few limitations should be mentioned. First, the sample was relatively small, and there was comparatively high dropout. In addition, the sample consisted of highly functional PLWH with good medical control of HIV infection, predominantly men. Furthermore, due to organizational reasons, the sample was diverse in terms of HIV-infection duration. It is therefore likely that other results would be obtained in a sample with a different gender ratio or/and clinical characteristics, especially concerning the stability of HRQoL. On the other hand, as was mentioned before, our participants were rather homogenous with regard to socio-medical variables, and dropout was a random factor that should not be viewed as systematic selection bias. Furthermore, we observed high variability in variance structure decomposition of HRQoL, as well as the high percentage of remaining variance in its trait component - this should be the subject of further studies. Finally, we used WHOQOL-BREF instead of WHOQOL-HIV-BREF, as at the time of conducting this study there was no Polish version of the WHOQOL-HIV-BREF. Nevertheless, WHOQOL-BREF was also used extensively among HIV/AIDS population and proved to be a reliable and a valid instrument to assess HRQoL also in this patient group [76–78].

## Conclusions

There is a consistency in HRQoL among PLWH, but also substantial occasion and method specificity that also vary between domains and within time. Specifically, the social domain indicator in WHOQQL-BREF can be regarded as unreliable, which should be considered when using the tool. Conversely, psychological domain is the most consistent, therefore it represents mainly stable personal disposition. In addition, HRQoL among PLWH is rather distinct from personality and socio-medical data, i.e., personality traits and socio-medical data altogether explained 26% of variance in the HRQoL trait component, indicating a uniqueness of HRQoL assessment in clinical practice. Thus, there is a need for more in-depth analyses of HRQoL evaluations to cover these patients’ substantial individual differences that are not attributable to their personality, medical, or socio-demographic characteristics.

## Abbreviations

HRQoL: Health-related quality of life
LGC: Latent growth-curve
LST: Latent state-trait
MCAR: Missing Completely at Random
ML: Maximum likelihood
PLWH: People living with HIV
PWB: Psychological well-being
QoL: Quality of life
WHO: World Health Organization
WHOQOL-BREF: WHO Quality of Life-BREF
